# Identification of residues crucial for the interaction between human neuroglobin and the α-subunit of heterotrimeric G_i_ protein

**DOI:** 10.1038/srep24948

**Published:** 2016-04-25

**Authors:** Nozomu Takahashi, Keisuke Wakasugi

**Affiliations:** 1 Department of Life Sciences, Graduate School of Arts and Sciences, The University of Tokyo, 3-8-1 Komaba, Meguro-ku, Tokyo 153-8902, Japan

## Abstract

Mammalian neuroglobin (Ngb) protects neuronal cells under conditions of oxidative stress. We previously showed that human Ngb acts as a guanine nucleotide dissociation inhibitor (GDI) for the α-subunits of heterotrimeric G_i/o_ proteins and inhibits the decrease in cAMP concentration, leading to protection against cell death. In the present study, we used an eukaryotic expression vector driving high-level expression of human wild-type Ngb or Ngb mutants that either exhibit or lack GDI activities in human cells. We demonstrate that the GDI activity of human Ngb is tightly correlated with its neuroprotective activity. We further demonstrate that Glu53, Glu60, and Glu118 of human Ngb are crucial for both the neuroprotective activity and interaction with Gα_i1_. Moreover, we show that Lys46, Lys70, Arg208, Lys209, and Lys210 residues of Gα_i1_ are important for binding to human Ngb. We propose a molecular docking model of the complex between human Ngb and Gα_i1_.

Neuroglobin (Ngb) is a globin widely expressed in the brain and which binds reversibly to oxygen (O_2_)^1^,^2^,^3^,^4^,^5^,^6^. Mammalian Ngb proteins can protect neurons from hypoxic-ischemic insults and protect the brain from experimentally induced stroke *in vivo*^7^,^8^,^9^,^10^,^11^. To investigate the neuroprotective mechanism of human Ngb under conditions of oxidative stress, we previously performed yeast two-hybrid screening using human Ngb as a bait and identified flotillin-1, a lipid raft microdomain-associated protein, as a binding partner^12^. We demonstrated that human Ngb is recruited to lipid rafts by interacting with flotillin-1 only during oxidative stress and that lipid rafts are crucial for neuroprotection by Ngb^11^. Moreover, we found that human ferric Ngb, which is generated under oxidative stress conditions, binds exclusively to the GDP-bound form of the α-subunits of heterotrimeric G_i/o_ proteins (Gα_i/o_), which are present in lipid rafts and inhibit adenylate cyclase activity^13^, thereby acting as guanine nucleotide dissociation inhibitor (GDI) for Gα_i/o_ and inhibiting the reduction of intracellular cAMP concentration to protect against cell death^10^,^11^,^14^,^15^,^16^.

Although Ngb was originally identified in mammalian species, it is also present in non-mammalian vertebrates^17^,^18^. We found that zebrafish Ngb does not exhibit GDI activity and cannot protect mammalian or zebrafish cells against oxidative stress-induced cell death^10^,^15^,^19^,^20^. In order to identify residues of human Ngb that are crucial for its GDI and neuroprotective activities, we prepared human Ngb mutants with a focus on residues differing between human and zebrafish Ngb and on exposed residues with positive or negative charges on the protein surface^15^. Protein transfection was achieved by using the protein delivery reagent Chariot, which can efficiently deliver a variety of proteins into several cell lines in a fully biologically active form^10^,^11^,^16^,^19^,^21^,^22^. We showed that human E53Q, R97Q, E118Q, and E151N Ngb mutants, which did not function as GDI proteins, did not rescue cell death under oxidative stress conditions^10^,^15^, indicating that Glu53, Arg97, Glu118 and Glu151 of human Ngb are crucial residues for its GDI activity and that the GDI activity of human Ngb is tightly correlated with its neuroprotective activity. The analysis using E60Q Ngb mutant revealed that Glu60 is also an essential residue for GDI and neuroprotective activities^16^. Furthermore, matrix-assisted laser desorption/ionization time-of-flight (MALDI-TOF) mass spectrometry (MS) analysis of tryptic peptides derived from a cross-linked complex between human wild-type (WT) Ngb and Gα_i1_, which is a member of the Gα_i/o_ family^13^, revealed cross-linking between Glu60 (Ngb) and Ser206 (Gα_i1_), and between Glu53 (Ngb) and Ser44 (Gα_i1_)^23^.

In the present study, in order to reconfirm the results using the protein transfection reagent “Chariot”, we used an eukaryotic expression vector which can provide the ability for high-level expression of human WT Ngb or Ngb mutants with or without GDI activities in human cells. We demonstrate that the GDI activity of human Ngb is tightly correlated with its neuroprotective activity. We also prepared site-directed mutants of Ngb or Gα_i1_ and investigated neuroprotective activities and protein-protein interactions by performing glutathione S-transferase (GST) pull-down assays. Moreover, we propose a model of the complex between human Ngb and Gα_i1_ based on our experimental results.

## Results

### Neuroprotective and Gα_i1_-binding assays of E53Q, E60Q, R97Q, E118Q, and E151Q Ngb mutants

**Figure 1:**
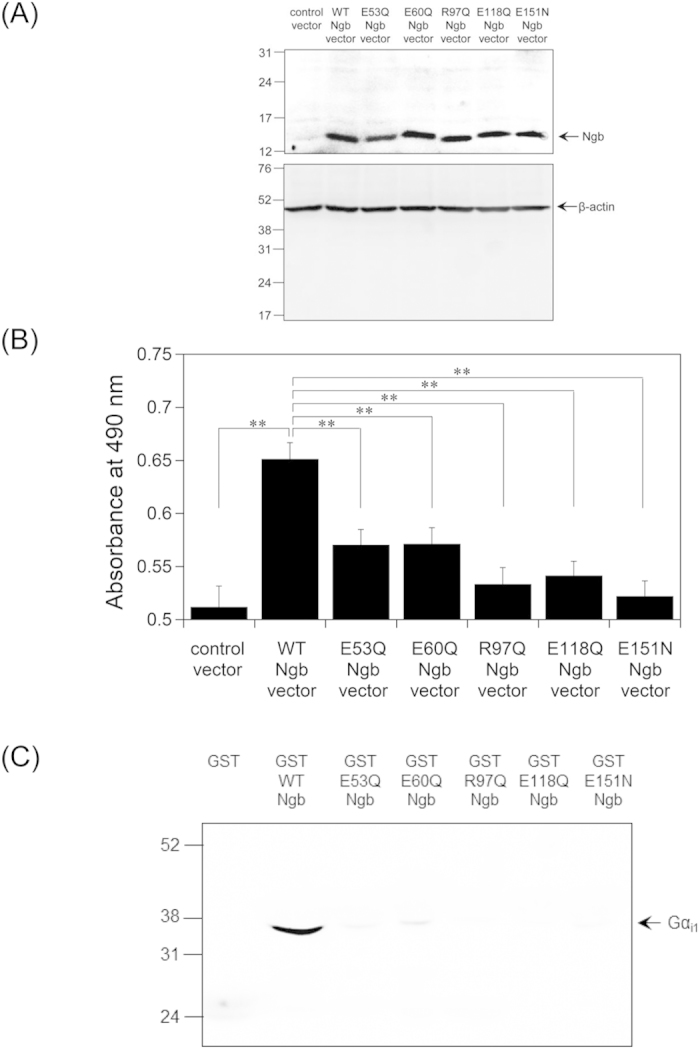
Effects of E53Q, E60Q, R97Q, E118Q, or E151N mutation in human Ngb on its neuroprotective activity and interaction with Gα_i1_. (**A**) Western blot analyses of SH-SY5Y cell lysates after transfection. Control vector, human WT or Ngb mutant expression vector was transfected into differentiated SH-SY5Y cells with Lipofectamine. The cells were then incubated for 24 h. Cell lysates were analyzed on 15.0% or 12.5% polyacrylamide-SDS gels and by Western blot analyses using rabbit anti-Ngb polyclonal antibody or mouse anti-β-actin monoclonal antibody, respectively. The arrow indicates the position expected for Ngb or β-actin. Molecular size markers (in kilodaltons) are shown at the left. (**B**) Effect of the mutation in human Ngb on SH-SY5Y cell death caused by hydrogen peroxide. Differentiated SH-SY5Y cells transfected with control vector, human WT or Ngb mutant expression vector with Lipofectamine were treated with hydrogen peroxide, and cell viability was measured by MTS assays. All data are expressed as means ± standard error of means (SEM) from four independent experiments, each carried out in triplicate. Data were analyzed by one-way ANOVA followed by Tukey-Kramer post hoc tests. **P < 0.01. (**C**) GST pull-down assays of human ferric WT Ngb or Ngb mutant with the GDP-bound truncated Gα_i1_. GST, GST-human WT Ngb, or GST-human Ngb mutant was incubated with human GDP-bound Gα_i1_ in a buffer (pH 7.4). Western blot analyses were performed with anti-Gα_i1_ mouse monoclonal antibody. The arrow indicates the position expected for Gα_i1_. Molecular size markers (in kilodaltons) are shown at the left.

We used SH-SY5Y cells differentiated into a neuron-like type to investigate the neuroprotective mechanism of human Ngb under oxidative stress conditions. A pcDNA3.1-human WT or Ngb mutant expression vector, or a control vector (pcDNA3.1 empty vector) was transfected into SH-SY5Y cells by Lipofectamine and the protective effects of Ngb proteins against hydrogen peroxide-induced cell death was tested. Expression of human Ngb proteins was confirmed by Western blot analyses (Fig. 1A). Cell viability was measured by using 3-(4,5-dimethylthiazol-2-yl)-5-(3-carboxymethoxyphenyl)-2-(4-sulfophenyl)-2H-tetrazolium, inner salt (MTS). The absorbance at 490 nm was directly proportional to the number of living cells (Supplementary Fig. S1). As shown in Fig. 1B, MTS assays showed that human WT Ngb enhanced cell survival. By contrast, human E53Q, E60Q, R97Q, E118Q, and E151N Ngb single mutants, which lack GDI activity, did not protect SH-SY5Y cells against cell death (Fig. 1B). These results are consistent with previous results using the protein transfection reagent “Chariot”^10^,^16^. Taken together, we reconfirmed that the GDI activity of human Ngb is tightly correlated with its neuroprotective activity.

Next, to characterize the protein-protein interaction between human Ngb and Gα_i1_ *in vitro*, we performed GST pull-down assays employing human Ngb fused to GST (GST-human Ngb). We previously demonstrated that GST-human ferric Ngb bound to the GDP-bound form of Gα_i1_^11^,^24^. These data are consistent with those obtained by surface plasmon resonance of non-tagged Ngb^14^, suggesting that the GST tag has no effect on protein-protein interactions between Ngb and Gα_i1_. In the present study, because the truncated Gα_i1_ protein, which lacks the N-terminal disordered region (25 amino acids), was more stable than the full-length enzyme^25^,^26^, GST-human ferric Ngb, or GST was purified (Supplementary Fig. S2), incubated with human truncated Gα_i1_ for GST pull-down assays and Western blot analyses were performed using antibody against Gα_i1_. As shown in Fig. 1C, GST-human WT Ngb bound to the GDP-bound form of the truncated Gα_i1_. Moreover, we found that human E53Q, E60Q, R97Q, E118Q, and E151N Ngb mutants did not, suggesting that Glu53, Glu60, Arg97, Glu118 and Glu151 residues of human Ngb are all indispensable for its interaction with Gα_i1_.

### Analyses based on a possible model of the complex between human Ngb and Gα_i1_

**Figure 2:**
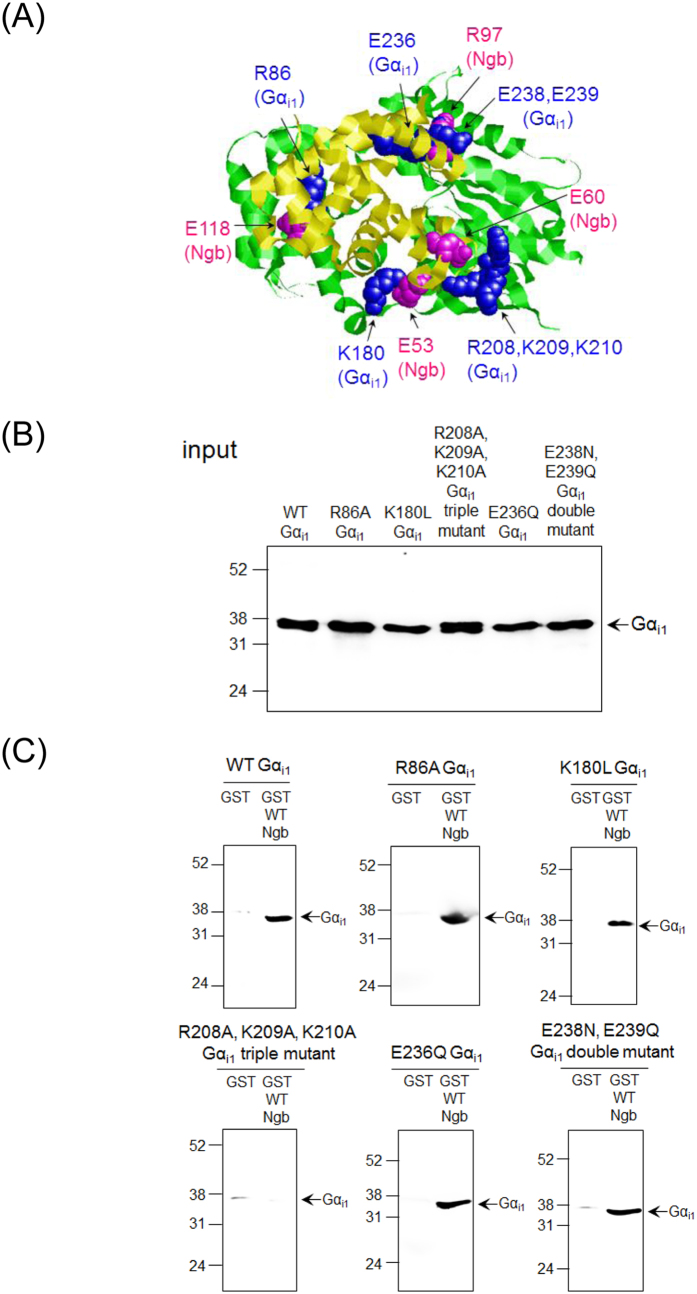
Effects of R86A, K180L, R208A, K209A, K210A, E236Q, E238N, or E239Q mutation in human Gα_i1_ on its interaction with Ngb. (**A**) A molecular docking modeling of the complex of Ngb and Gα_i1_. Tertiary structure of human Ngb (Protein Data Bank code: 4MPM) is highlighted in yellow. Residues in human Ngb crucial for its neuroprotective activity are indicated in magenta. Tertiary structure of human Gα_i1_, which binds to the GoLoco motif of RGS14 (Protein Data Bank code: 1KJY), is highlighted in green. Residues in Gα_i1_ are indicated in blue. (**B,C**) GST pull-down assays of human ferric WT Ngb with the GDP-bound form of truncated WT Gα_i1_, R86A, K180L, or E236Q Gα_i1_ single mutant, E238N, E239Q Gα_i1_ double mutant, or R208A, K209A, K210A Gα_i1_ triple mutant. GDP-bound WT Gα_i1_ or Gα_i1_ mutant was incubated with GST-human Ngb or GST in a buffer (pH 7.4). Western blot analyses of the input (**B**) and pull-down samples (**C**) were performed with anti-Gα_i1_ mouse monoclonal antibody. The arrows indicate the positions expected for Gα_i1_. Molecular size markers (in kilodaltons) are shown at the left.

To gain further insight into the structure of the complex between human Ngb and Gα_i1_, we created a molecular docking model of the complex based on the following two experimental results: i) Glu53, Glu60, Arg97, Glu118, and Glu151 of human Ngb are all crucial residues for its neuroprotective effect and interaction with Gα_i1_; ii) Glu53 and Glu60 of human Ngb were cross-linked to Ser44 and Ser206 of Gα_i1_, respectively^23^. The molecular docking model was created by manually adjusting the strucutres of Ngb and Gα_i1_ to avoid steric hindrance between Ngb and Gα_i1_ and is shown in Fig. 2A.

Next, to evaluate this model, we prepared site-directed Gα_i1_ mutants at residues with positive or negative charges at the protein binding interface between human Gα_i1_ and Ngb: R86A, K180L, and E236Q Gα_i1_ single mutants, an E238N, E239Q Gα_i1_ double mutant, and a R208A, K209A, K210A Gα_i1_ triple mutant. For example, Lys180 of human Gα_i1_ was mutated to Leu because the corresponding residue of Lys180 of Gα_i1_ in human Gα_s_ is Leu. An E238N, E239Q Gα_i1_ double mutant was generated because the corresponding residues of Glu238 and Glu239 of Gα_i1_ in human Gα_s_ are Asn and Gln, respectively. As shown in Fig. 2B,C, R208A, K209A, K210A Gα_i1_ triple mutant did not interact with human ferric WT Ngb, whereas R86A, K180L, and E236Q Gα_i1_ single mutants, and the E238N, E239Q Gα_i1_ double mutant bound to Ngb to an extent similar to that of WT Gα_i1_. These data suggest that Arg208, Lys209, Lys210, but not Arg86, Lys180, Glu236, Glu238, Glu239, of Gα_i1_ are crucial for the interaction with Ngb and that the binding model in Fig. 2A is not appropriate.

### Functional analyses of Ngb mutants prepared on the basis of amino acid sequence alignment among various species of Ngb

**Figure 3:**
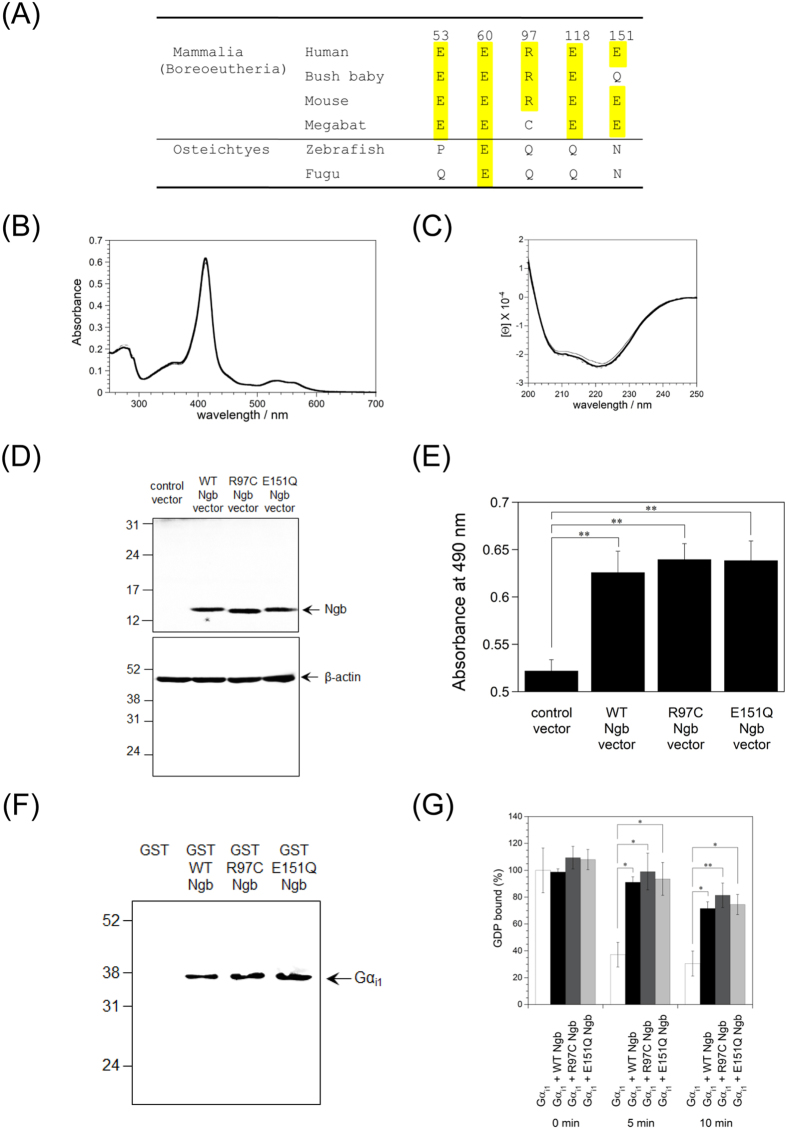
Effects of R97C or E151Q mutation in human Ngb on its structure, GDI and neuroprotective activities. (**A**) Partial sequence alignment among human, bush baby, mouse, megabat, zebrafish and fugu Ngb proteins. Identical residues to human Ngb are highlighted in yellow. Numbers above the sequences correspond to those of the residues of human Ngb. (**B**) Electronic absorption spectra of the ferric form of human WT Ngb (bold line), R97C Ngb (fine line), and E151Q Ngb (dotted line). (**C**) CD spectra in the far UV region of the ferric form of human WT Ngb (bold line), R97C Ngb (fine line), and E151Q Ngb (dotted line). (**D**) Western blot analyses of SH-SY5Y cell lysates after transfection. Control vector, human WT or Ngb mutant expression vector was transfected into differentiated SH-SY5Y cells with Lipofectamine. The cells were then incubated for 24 h. Cell lysates were analyzed on 15.0% or 12.5% polyacrylamide-SDS gels and by Western blot analyses using rabbit anti-Ngb polyclonal antibody or mouse anti-β-actin monoclonal antibody, respectively. The arrow indicates the position expected for Ngb or β-actin. Molecular size markers (in kilodaltons) are shown at the left. (**E**) Effect of the mutation in human Ngb on SH-SY5Y cell death caused by hydrogen peroxide. Differentiated SH-SY5Y cells transfected with control vector, human WT or Ngb mutant expression vector with Lipofectamine were treated with hydrogen peroxide, and cell viability was measured by MTS assay. All data are expressed as means ± SEM from four independent experiments, each carried out in triplicate. Data were analyzed by one-way ANOVA followed by Tukey-Kramer post hoc tests. **P < 0.01. (**F**) GST pull-down assays of the human Ngb mutant with GDP-bound Gα_i1_. GST, GST-human WT Ngb, or GST-human Ngb mutant was incubated with human GDP-bound Gα_i1_ in a buffer (pH 7.4). Western blot analyses were performed with anti-Gα_i1_ mouse monoclonal antibody. The arrow indicates the position expected for Gα_i1_. Molecular size markers (in kilodaltons) are shown at the left. (**G**) The effect of the R97C or E151Q mutation on dissociation of GDP from human GDP-bound Gα_i1_. The amount of [^3^H]GDP bound to Gα_i1_ in the absence of human ferric Ngb at 0 min was defined as 100%. All data are expressed as means ± SEM from three independent experiments. Data were analyzed by one-way ANOVA followed by Tukey-Kramer post hoc tests. *P < 0.05, **P < 0.01.

We previously reported that Glu53, Arg97, Glu118, and Glu151 of human Ngb are conserved only among boreoeutheria of mammalia, and not among afrotheria, maetatheria, prototheria, aves, reptillia, amphibia, or osteichtyes^27^, suggesting that boreoeutheria Ngb proteins may protect neurons against oxidative stress-induced cell death. In the present study, we performed more detailed sequence comparisons by using sequence data of Ngb proteins from a greater number of species. As shown in Fig. 3A and Supplementary Fig. S3, the residue corresponding to Arg97 of human Ngb is a Cys in megabat Ngb. And the residue corresponding to Glu151 of human Ngb is a Gln in bush baby Ngb (Fig. 3A and Supplementary Fig. S3). Therefore, we created human R97C and E151Q Ngb mutants and investigated their structure, neuroprotective activity and interaction with Gα_i1_.

Initially, we evaluated the effects of the R97C or E151Q mutation on the electronic state of the heme group by measuring the absorption spectra. As shown in Fig. 3B and Table 1, the wavelength of the Soret peak of human ferric R97C or E151Q Ngb was the same as those of human ferric WT Ngb, demonstrating that the R97C or E151Q mutation of human Ngb did not perturb the electric state of the heme group. Next, to examine the effect of the R97C or E151Q substitution upon secondary structure, we measured the far-UV circular dichroism (CD) spectra. As shown in Fig. 3C, human WT, R97C and E151Q Ngb proteins exhibited two negative broad peaks around 222 and 208 nm, which are characteristic of an α-helical structure. The α-helical contents of the R97C and E151Q Ngb proteins were estimated to be 68.9% and 73.4%, which is almost identical to that of human WT Ngb (71.4%) (Table 1). These results showed that the secondary protein structure is not affected by the amino acid substitution.

Next, we tested whether R97C and E151Q Ngb can protect SH-SY5Y cells against oxidative stress. We first confirmed that human WT, R97C, and E151Q Ngb proteins were expressed at a similar level in SH-SY5Y cells by Western blot analyses (Fig. 3D). MTS assays showed that the expression of human R97C or E151Q Ngb mutant in the cells rescued cell death under oxidative stress condition, as did that of human WT Ngb (Fig. 3E). Moreover, GST-human ferric Ngb, or GST was purified (Supplementary Fig. S2), incubated with human truncated Gα_i1_ and Western blot analyses were performed using antibody against Gα_i1_. As shown in Fig. 3F, GST pull-down assays showed that R97C and E151Q Ngb bound to the truncated Gα_i1_ as did the WT Ngb. Furthermore, to examine the effect of the R97C or E151Q mutation of human Ngb upon the release of GDP from Gα_i1_, we measured the rates of GDP dissociation in the absence or presence of human Ngb. In the presence of an excess amount of unlabeled GTP, [^3^H]GDP release from [^3^H]GDP-bound Gα_i1_ was inhibited by human ferric WT Ngb (Fig. 3G). As shown in Fig. 3G, human ferric R97C and E151Q Ngb functioned as the GDI for Gα_i1_ as did human WT Ngb. These results suggest that the positive charge of Arg97 and the negative charge of Glu151 of human Ngb are not essential for the GDI and neuroprotective activities. Taken together, we conclude that Glu53, Glu60, and Glu118 of human Ngb are the only crucial residues for its activities.

### Search for residues of Gα_i1_ crucial for the interaction with human Ngb

**Figure 4:**
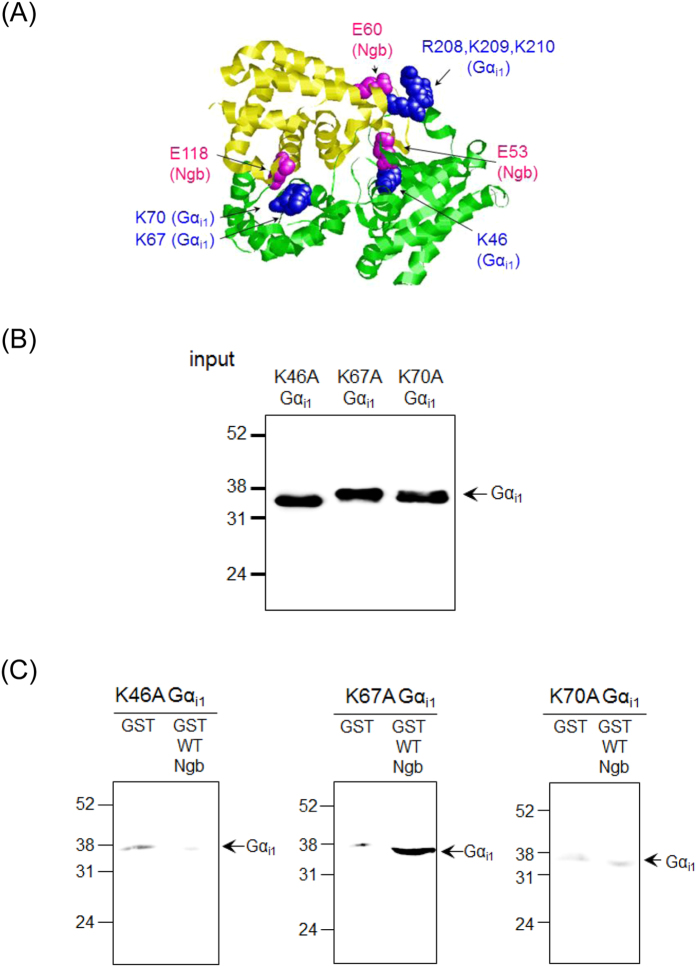
Effects of K46A, K67A, or K70A mutation in human Gα_i1_ on its interaction with Ngb. (**A**) A molecular docking model of the complex of Ngb and Gα_i1_. Tertiary structure of human Ngb (Protein Data Bank code: 4MPM) is highlighted in yellow. Residues in human Ngb crucial for its neuroprotective activity are indicated in magenta. Tertiary structure of human Gα_i1_, which binds to the GoLoco motif of RGS14 (Protein Data Bank code: 1KJY), is highlighted in green. Residues in Gα_i1_ are indicated in blue. (**B,C**) GST pull-down assays of human ferric WT Ngb with the GDP-bound form of truncated WT Gα_i1_, K46A, K67A, or K70A Gα_i1_ single mutant. GDP-bound form of WT Gα_i1_ or Gα_i1_ mutant was incubated with GST-human Ngb or GST in a buffer (pH 7.4). Western blot analyses of the input (**B**) and pull-down samples (**C**) were performed with anti-Gα_i1_ mouse monoclonal antibody. The arrows indicate the positions expected for Gα_i1_. Molecular size markers (in kilodaltons) are shown at the left.

Next, because we found that Arg97 and Glu151 residues of Ngb are not involved in the interface with Gα_i1_, we created a new molecular docking model of the complex between human Ngb and Gα_i1_, which is depicted in Fig. 4A. To clarify binding sites in the complex between human Ngb and Gα_i1_, we prepared K46A, K67A, and K70A Gα_i1_ single mutants. As shown in Fig. 4B,C, K46A and K70A Gα_i1_ single mutants did not interact with Ngb. Moreover, the R208A, K209A, K210A Gα_i1_ triple mutant also did not interact with Ngb (Fig. 2B,C). These results suggest that the model of Fig. 4A is correct. Moreover, K67A Gα_i1_ bound to Ngb as did WT Gα_i1_ (Fig. 4B,C), indicating that Glu118 of human Ngb interacts with Lys70, but not Lys67, of Gα_i1_ in a site-specific manner.

## Discussion

In the present study, by using eukaryotic expression vectors for human Ngb proteins we demonstrated that the GDI activity of human Ngb is tightly correlated with its neuroprotective activity. GST pull-down assays using GST-fused human Ngb demonstrated that Glu53, Glu60, Arg97, Glu118 and Glu151 residues of human Ngb are all indispensable for its interaction with Gα_i1_. However, R97C and E151Q Ngb single mutants, generated based on sequence alignment among various species of Ngb, showed that the positive charge of Arg97 and the negative charge of Glu151 of human Ngb are dispensable for both the GDI and neuroprotective activities. We therefore speculated that the lack of GDI and neuroprotective activities of the R97Q and E151N Ngb mutants may arise from structural alterations induced by the mutations and concluded that only the negative charges of Glu53, Glu60, and Glu118 of human Ngb are crucial for the GDI and neuroprotective activities. We measured the UV-visible and CD spectra of the WT and mutants but could not detect any significant structural differences among them. Further studies are in progress to clarify structural alterations between the WT and R97Q or E151N Ngb mutant.

Moreover, we identified residues of Gα_i1_ crucial for the interaction with human Ngb; Lys46, Lys70, Arg208, Lys209, and Lys210 residues of Gα_i1_ are important for its binding to human Ngb. The present results imply that electrostatic interactions between the negative charges of Glu53, Glu60, and Glu118 of human Ngb and the positive charges of Lys46, Lys70, Arg208, Lys209, and/or Lys210 of Gα_i1_ are crucial for the formation of a complex between Ngb and Gα_i1_. This is consistent with the previous observation that electrostatic complementarity is an important factor for the interaction of Gα with its regulator^28^. Further more detailed studies are in progress to investigate the significance of Arg208, Lys209, and/or Lys210 of Gα_i1_. Moreover, because the residue corresponding to Arg97 of human Ngb is a Gln in yak Ngb (Supplementary Fig. S3), it is interesting to investigate whether yak Ngb has the GDI and neuroprotective activities.

The present results support that a molecular docking model of the complex between human Ngb and Gα_i1_ in Fig. 4A. In this model, Glu60 (Ngb), which was cross-linked to Ser206 (Gα_i1_) by chemical cross-linking^23^, interacts with Arg208, Lys209, and/or Lys210 of Gα_i1_. The amino acid sequence surrounding Glu60 in human Ngb has a motif homologous to those of the R6A-1 peptide and the KB-752 peptide, which interact with GDP-bound Gα_i1_^16^. It has previously been reported that the R6A-1 and KB-752 peptides interact with the switch II (a.a. 199–219) of Gα_i1_^26^,^29^,^30^. The molecular docking model of the complex between human Ngb and Gα_i1_ shows that Glu60 of human Ngb is located near the switch II of Gα_i1_, as are R6A-1 and KB-752 peptides, suggesting that the motif including Glu60 in human Ngb functions as the core motif for the binding with Gα_i1_.

As shown in Fig. 4A, Glu53 (Ngb), which was cross-linked to Ser44 (Gα_i1_) by chemical cross-linking^23^, binds to Lys46 of Gα_i1_. X-ray structural data of Gα_i1_ show that Ser44 and Lys46 of Gα_i1_ are located in the vicinity of GDP^25^. This implies that Ngb could be positioned near the GDP-binding site in the Ngb-Gα_i_ complex, which would inhibit the dissociation of GDP from the binding site. Moreover, it should be also noted that Glu53 of Ngb is conserved among boreotheria except for the harp or hooded seal (Supplementary Fig. S3). In seals Ngb is mainly localized in astrocytes, whereas other boreotheria Ngb exists in neuron^31^,^32^. The different localization of Ngb suggests that the function of seal Ngb may be different from that of other boreotheria Ngb.

Figure 4A shows that Glu118 of Ngb interacts with Lys70 of Gα_i1_. We previously carried out chemical cross-linking of the Ngb and Gα_i1_ complex by treatment with zero-length cross-linkers to identify the sites of interaction between Ngb and Gα_i1_^23^. In the peptide map of tryptic peptides derived from the cross-linked Ngb-Gα_i1_ complex, the MS peak of a Ngb peptide (amino acids 103–119 of Ngb), which was observed in the tryptic peptide map of Ngb, was missing or significantly decreased in intensity^23^, suggesting that the 103–119 amino acid residues of Ngb are involved in the Ngb-binding site for Gα_i1_, but information about the detailed residues cross-linked in the complex of Ngb and Gα_i1_ remained unclear. The present results suggest that Glu118, which is located in the 103–119 region, of human Ngb interacts with Lys70 residue, which is located in the α-helical domain of Gα_i1_, as shown in Fig. 4A. Strikingly, crystal structure of GoLoco motifs of LGN protein, which act as GDI for Gα_i1_, elucidated that they interact with the switch II region and Tyr69 and Val72, which are close to Lys70, in the α-helical domain of Gα_i1_^33^. It is also worth noting that RGS domains, which bind to Gα_i_ selectively, interact with some residues in the α-helical domain of Gα as well as the switch regions^28^.

Lys46, Arg208, Lys209, and Lys210 residues, which are crucial for the interaction with Ngb, and their sequential neighborhoods of Gα_i1_ are conserved among Gα_i/o_ and Gα_s_. In contrast, Lys70 residue of Gα_i1_ is conserved among Gα_i/o_, but corresponds to Val in Gα_s_ based on sequence alignment among members of the Gα family^13^. Moreover, sequential neighborhoods around Lys70 of Gα_i/o_ are very different from those of Gα_s_. Therefore, the positive charge of the 70^th^ residue of Gα, which interacts with the negative charge of carboxylic group of Glu118 through electrostatic interaction, may be crucial for the Gα_i/o_–specific binding property of Ngb.

## Methods

### Cell culture

SH-SY5Y cells (CRL-2266) were obtained from the American Type Culture Collection (ATCC; Manassas, VA) and maintained in a 1:1 mixture of Dulbecco’s modified Eagle’s medium and Ham’s F-12 nutrient mixture containing 2.5 mM glutamine, supplemented with 10% (v/v) fetal bovine serum, 100 U/ml penicillin, and 100 μg/ml streptomycin (all from Invitrogen, Carlsbad, CA) in a humidified atmosphere containing 5% CO_2_ at 37 °C. The medium was changed every 4 days, and the cultures were split at a 1:20 ratio once a week. Cultured cells were induced to differentiate into a neuronal phenotype by treatment with 10 μM retinoic acid (Sigma-Aldrich, St. Louis, MO) over a period of 6 days (media were exchanged every 3 days during sub-culture). Differentiation was verified by monitoring macroscopic changes to the cells.

### Transfection of Ngb expression vector into SH-SY5Y cells and treatment of cells with hydrogen peroxide

The eukaryotic expression vector pcDNA3.1 (Invitrogen) for human Ngb was prepared as described previously^11^. A QuikChange^TM^ site-directed mutagenesis system (Stratagene, La Jolla, CA) was used for site-directed mutagenesis and the constructs were confirmed by DNA sequencing (FASMAC Co., Ltd., DNA sequencing services, Atsugi, Japan). Differentiated SH-SY5Y cells were plated on poly-D-lysine coated 96-well plates at a density of 5.0 × 10^5^ cells/mL for 24 h. The pcDNA3.1-human WT or Ngb mutant expression vector or control vector (pcDNA3.1 empty vector) was transfected by using Lipofectamine^TM^ 2000 (Invitrogen) according to the manufacturer’s instructions. After 24 h of transfection, hydrogen peroxide (100 μM) was added and cells were incubated for 24 h.

### Western blot analyses

After cell lysates were resolved by electrophoresis on polyacrylamide-SDS gels, proteins were electroblotted onto Hybond-P PVDF membranes (GE Healthcare Biosciences, Piscataway, NJ), which were then blocked with phosphate-buffered saline (PBS) and 5% skim milk (Wako Pure Chemical Industries, Osaka, Japan) and incubated with rabbit anti-Ngb (FL-151) polyclonal antibody (Santa Cruz Biotechnology, Santa Cruz, CA), or mouse anti-β-actin monoclonal antibody (Sigma-Aldrich). After washing, membranes were incubated with an HRP-linked F(ab′)_2_ fragment of donkey anti-rabbit IgG or an HRP-linked whole antibody of sheep anti-mouse IgG (GE Healthcare Biosciences). Proteins were visualized using ECL^TM^ Western blotting detection reagents (GE Healthcare Biosciences). Chemiluminescent signals were detected using a LAS-4000 mini luminescent image analyzer (GE Healthcare Biosciences).

### Cell viability assay

Cell viability was measured with the CellTiter 96^®^ AQueous One Solution Cell Proliferation Assay Reagent (Promega, Madison, WI), containing [3-(4,5-dimethylthiazol-2-yl)-5-(3-carboxymethoxyphenyl)-2-(4-sulfophenyl)-2H-tetrazolium, inner salt; MTS]. Cultured cells were incubated with the MTS reagent at 37 °C for 4 h in a humidified, 5% CO_2_ atmosphere. The amount of colored formazan dye formed was then quantified by measuring absorbance at 490 nm with a Beckman Coulter DTX880 plate reader (Beckman Coulter, Fullerton, CA).

### Preparation and purification of GST and a fusion protein of GST and Ngb

Human Ngb cDNA was cloned into the pGEX-4T-1 vector (GE Healthcare Biosciences) to produce the fusion protein GST-Ngb^11^,^24^. A QuikChange^TM^ site-directed mutagenesis system (Stratagene) was used for site-directed mutagenesis. The constructs were confirmed by DNA sequencing (FASMAC Co., Ltd., DNA sequencing services). Overexpression of GST-Ngb and GST alone (as a control) was induced in the *Escherichia coli* strain BL21 (DE3) (Novagen, Madison, WI) by treatment with isopropyl-β-D-thiogalactopyranoside (IPTG) for 4 h. Both GST-ferric Ngb and GST were purified by using glutathione-Sepharose 4B beads (GE Healthcare Biosciences) according to the manufacturer’s instructions.

### Preparation of recombinant human truncated Gα_i1_ protein

The DNA fragment containing the human truncated Gα_i1_ subunit (residues 26–354) was amplified by PCR and cloned into the pET151/D-TOPO^®^ vector (Invitrogen) to be expressed as human WT truncated Gα_i1_ protein (residues 26–354) fused to a TEV protease recognition site directly after an N-terminal tag of six histidine residues (His_6_-tag). A QuikChange^TM^ site-directed mutagenesis system (Stratagene) was used for site-directed mutagenesis. The constructs were confirmed by DNA sequencing (FASMAC Co., Ltd., DNA sequencing services). The resulting Gα_i1_ was expressed in *E. coli* strain BL21 (DE3) by induction with IPTG and purified by using a nickel affinity column (His·Bind^®^ resin; Novagen), as described previously^11^. Then, the sample was incubated with His_6_-tagged TEV protease (MoBiTec GmbH, Göttingen, Germany) and loaded onto a His·Bind^®^ column to separate the cleaved Gα_i1_ from the cleaved His_6_-tag, any uncleaved protein, and His_6_-tagged TEV protease, as described previously^11^.

### GST pull-down assays using truncated Gα_i1_

Truncated Gα_i1_ was incubated with either GST alone or GST–ferric Ngb immobilized on glutathione-Sepharose 4B beads (GE Healthcare Biosciences) in HEPES buffer (10 mM HEPES, 150 mM NaCl, 10 mM MgCl_2_, 10 μM GDP, 0.1% Tween20, pH 7.4) for 1 h at 4 °C. The beads were washed extensively three times with the buffer, and the samples were then resuspended in Laemmli sample buffer, heated for 5 min at 95 °C, and separated on 12.0% polyacrylamide-SDS gels. For Western blot analyses, the proteins were transferred onto Hybond-P PVDF membranes (GE Healthcare Biosciences), which were then blocked with PBS and 5% skim milk (Wako Pure Chemical Industries) and incubated with mouse anti-Gα_i1_ (Ab-3; clone R4.5) monoclonal antibody (Thermo Fisher Scientific, Fremont, CA). After washing, the membranes were incubated with an HRP-linked whole antibody of sheep anti-mouse IgG (GE Healthcare Biosciences). Proteins were visualized using ECL^TM^ western blotting detection reagents (GE Healthcare Biosciences). Chemiluminescent signals were detected using a LAS-4000 mini luminescent image analyzer (GE Healthcare Biosciences).

### Preparation of non-tagged recombinant human Ngb proteins

Plasmids for human Ngb were prepared as described previously^14^,^15^. A QuikChange^TM^ site-directed mutagenesis system (Stratagene) was used for site-directed mutagenesis. The constructs were confirmed by DNA sequencing (FASMAC Co., Ltd., DNA sequencing services). Overexpression of each Ngb was induced in *E. coli* strain BL 21 (DE 3) by treatment with IPTG for 4 h, and each Ngb protein was purified as described previously^10^,^11^,^14^,^15^,^16^. Briefly, soluble cell extracts were loaded onto DEAE sepharose anion-exchange columns equilibrated with buffer A (20 mM Tris-HCl, pH 8.0). Ngb proteins were eluted from columns with buffer A containing 150 mM NaCl, and further purified by passage through Sephacryl S-200 HR gel filtration columns. The protein concentration of human ferric Ngb was determined spectrophotometrically using an extinction coefficient of 122 mM^−1^cm^−1^ at the Soret peak.

### UV-visible spectra

Electronic absorption spectra of purified proteins were recorded with a UV-visible spectrophotometer (UV-2450; Shimadzu, Kyoto, Japan) at ambient temperature (~20 °C). Spectra were recorded in PBS (pH 7.4).

### CD spectra

CD spectra in the far-UV region were measured with a spectropolarimeter (J-805; JASCO Co., Tokyo, Japan) at 20 °C. The samples were measured at a concentration of approximately 5 μM in 50 mM sodium phosphate buffer (pH 7.4). The path length of the cells used for the measurements was 1 mm. The molar ellipticity (deg cm^2^ dmol^−1^) was determined on the mean residue basis. The α-helix content (*f*_H_) was calculated according to Chen *et al.*^34^ by the following equation:


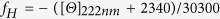


### [^3^H]GDP dissociation assays

GDP dissociation assays were performed, as described previously^11^,^16^. In brief, Gα_i1_ complexed with [^3^H]GDP (0.3 μM) was prepared by incubating 0.3 μM Gα_i1_ with 2 μM [8,5′-^3^H]GDP (PerkinElmer Life Sciences, Boston, MA) in buffer B [20 mM Tris-HCl, 100 mM NaCl and 10 mM MgSO_4_ at pH 8.0] for 1.5 h at 25 °C. Excess unlabeled GTP (2 mM) was added to monitor dissociation of [^3^H]GDP from Gα_i1_ in the absence or presence of non-tagged ferric Ngb (10 μM). Aliquots were withdrawn at 0, 5, and 10 min and were passed through nitrocellulose filters (0.45 μm) (Advantec Toyo, Tokyo, Japan). The filters were then washed three times with 1 ml of ice-cold buffer B and were counted in a liquid scintillation counter (LS6500; Beckman Coulter).

## Additional Information

**How to cite this article**: Takahashi, N. and Wakasugi, K. Identification of residues crucial for the interaction between human neuroglobin and the α-subunit of heterotrimeric G_i_ protein. *Sci. Rep.* **6**, 24948; doi: 10.1038/srep24948 (2016).

## Supplementary Material

Supplementary Figures S1, S2, S3

## Figures and Tables

**Table 1:** Structural data of human ferric WT Ngb and Ngb mutants.

	WT	WT	E53Q	E60Q	R97Q	R97C	E118Q	E151N	E151Q
*Electronic absorption spectra*									
Soret (nm)	413	413	413	413	413	413	413	413	413
*CD spectra*									
[*Θ*]_222_ _nm_ × (10^−4^) (deg cm^2^ dmol^−1^)	−2.32	−2.40	−2.40	−2.29	−2.26	−2.32	−2.34	−2.36	−2.46
α-helical content (%)	68.9	71.4	71.5	67.9	66.9	68.9	69.5	70.2	73.4
References	Ref. 16	this study	Ref. 16	Ref. 16	Ref. 16	this study	Ref. 16	Ref. 16	this study
