# Supplementary material for: Identification of residues crucial for the interaction between human neuroglobin and the α-subunit of heterotrimeric Gi protein
Source: Sci Rep. 2016 Apr 25;6:24948. doi: 10.1038/srep24948 (PMC4842972; doi:10.1038/srep24948)
Supplement: Supplementary Figures S1, S2, S3 [file srep24948-s1.pdf]

Supplementary Figures

Identification of residues crucial for the interaction between human neuroglobin and the α-subunit of heterotrimeric G_i protein

Nozomu Takahashi and Keisuke Wakasugi *

Department of Life Sciences, Graduate School of Arts and Sciences, The University of Tokyo, 3-8-1 Komaba, Meguro-ku, Tokyo 153-8902, Japan.

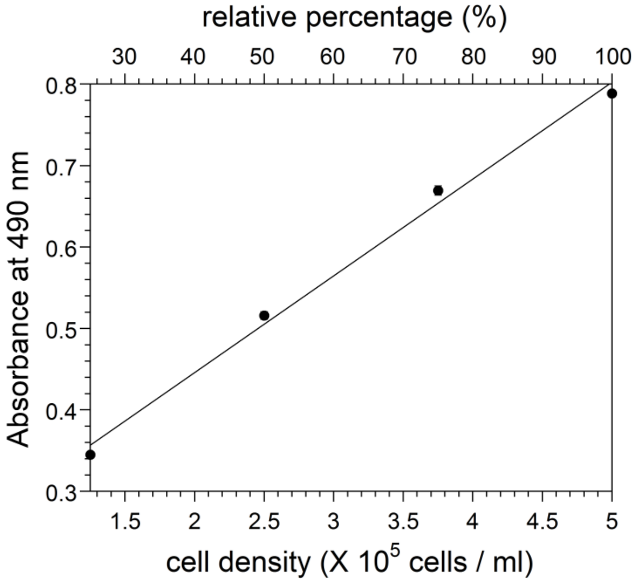

Supplementary Figure S1. Effect of cell density on absorbance at 490 nm measured using the MTS reagent. Differentiated SH-SY5Y cells were plated on poly-D-lysine coated 96-well plates at the indicated cell density. The cells were incubated for 72 h. Cultured cells were incubated with the MTS reagent for 4 h in a humidified, 5% CO_2 atmosphere. The amount of colored formazan dye formed was then quantified by measuring absorbance at 490 nm with a Beckman Coulter DTX880 plate reader (Beckman Coulter, Fullerton, CA). Each point represents the mean ± SEM from three independent experiments, each carried out in triplicate. The correlation coefficient of the line was 0.997, indicating a linear response between cell density and absorbance at 490 nm.

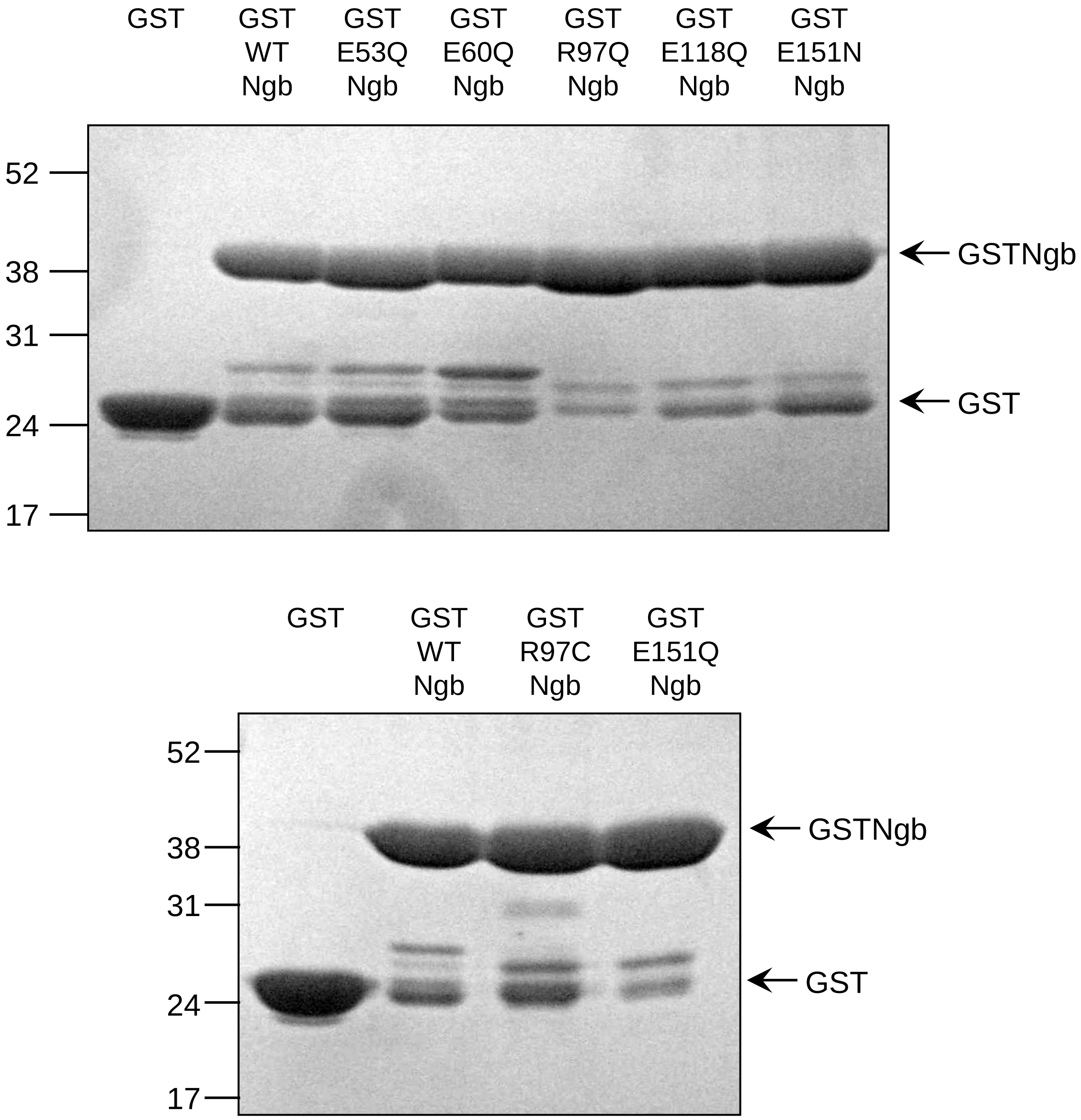

Supplementary Figure S2. SDS-polyacrylamide gel electrophoresis of GST, GST-human ferric WT Ngb, or GST-human ferric Ngb mutants used for GST pull-down assays. The samples were analyzed on a 12.0% SDS-polyacrylamide gel and stained with Coomassie Blue. Molecular size markers (in kilodaltons) are shown at the left.

				Sequence (residues 53, 60 highlighted)
Mammalia	Eutheria	Boreotheria	Human	-----MERPEPELIRQSWRAVSRSPLEHGTVLFARLFALEPDLLPLFQYNCRQFSSPEDCLSSPE
			Bush baby	-----MERPEPELIRQSWQAVSRSPLEHGTVLFARLFDLEPDLLPLFQYNCRQFSSPEDCLSSPE
			Yak	-----MELPEPELIRQSWREVSRSPLEHGTVLFARLFDLEPDLLPLFQYNCRQFSSPEDCLSSPE
			Mouse	-----MERPESELIRQSWRVVSRSPLEHGTVLFARLFALEPSLLPLFQYNGRQFSSPEDCLSSPE
			Megabat	-----MERPEP---SRWSWTVSRKPLEHGGRSAG-LFDLEPDLLPLFQYKSRQFSSHEDCLSSPE
			Whale	-----MERPEPELIRQSWREVSRSPLEHGTVLFARLFDLEPDLLPLFQYNCRQFSSSEDCLSSPE
			Harp seal	-----MERPEPELIRQSWREVSRSPLEHGTVLFARLFDLEPDLLPLFQYNCRQFSSPKDCLSSPE
			Hooded seal	-----MERPEPELIRQSWRAVSRSPLEHGTVLFARLFDLEPDLLPLFQYNCRQFSSPKDCLSSPE
		Afrotheria	Elephant	-----MQRPEHELIRQSWRVVSRSPLEHGTVLFARLFDLEPDLLPLFQYNCRQFSSVNDCLSSPE
	Metatheria		Opossum	METRRLSGPEQELIRESWQKVNSNPLQHGMILFTRLFDLEPDLLPLFQYNCRQFSSPQDCLSSPE
	Prototheria		Platypus	MENGRLSGPEQELIRESWRSVNSNPLEHGMILFTRLFDLEPDLLPLFQYNCRQFSSPRDCLASPE
Osteichtyes			Zebrafish	--MEKLSEKDKGLIRDSWESLGKNKVPHGIVLFTRLFELDPALLTLFSYSTN-CGDAPECLSSPE
			Fugu	--MEKLSSKDKELIRGSWDSLGKNKVPHGVIMFSRLFELDPELLSLFHYTTN-CGSTQDCLSSPE

	Sequence (residues 97, 118, 151 highlighted)
Human	FLDHIRKVMLVIDAAVTNVEDLSSLEEYLASLGRKHRAVGVKLSSFST----VGESLLYMLEKCLGPAFTPATRAAWSQLYGAVVQAMSRGWDGE------
Bush baby	FLDHIRKVMLVIDAAVTNVEDLSSLEEYLASLGRKHRAVGVKLSSFST----VGESLLYMLEKCLGPAFTPATRAAWSQLYQTVVQAMSRGWDGQ------
Yak	FLDHIRKVMLVIDAAVTNVEDLSSLEEYLAGLGRKHQAVGVKLSSFST----VGESLLYMLEKCLGPAFTPATRAAWSQLYGAVVQAMSRGWGGE------
Mouse	FLDHIRKVMLVIDAAVTNVEDLSSLEEYLTSLGRKHRAVGVRLSSFST----VGESLLYMLEKCLGPDFTPATRTAWSRLYGAVVQAMSRGWDGE------
Megabat	FLDHIRKVILVIDAAVTNVEDLSSLEEYLAGLGRKHCAVGVKLCFFSA----VGESLLYMLEKCPGPVFTPAVRAAWSQLYGAVVQAMSRGWDGE------
Whale	FLDHIRKVMLVIDAAVTNVEDLSSLEEYLASLGRKHRAVGVKLSSFST----VGESLLYMLEKCLGPAFTPAMRAAWSQLYGAVVQAMSRGWDGD------
Harp seal	FLDHIRKVMLVIDTAVTNVEDLSSLEEYLAGLGKKHRAVGVKLSSFST----VGESLLYMLEKCLGPAFTPAVRAAWSQLYAAVVQAMSRGWDGE------
Hooded seal	FLDHIRKVMLVIDTAVTNVEDLSSLEEYLAGLGKKHRAVGVKLSSFST----VGESLLYMLEKCLGPAFTPAVRAAWSQLYAAVVEAMSRGWDGE------
Elephant	FLDHIRKVMVVIDAAVTNVEDLSSLEEYLAGLGKKHRLVGVKLSSFSVGTETVGESLLYMLEKCLGPAFTPATKAAWSQLYGAVVQAMSRGWDGE------
Opossum	FLDHIRKVMLVIDAAVTHVENLSSLEEYLTNLGKKHKAVGVKLSSFST----VGESLLYMLEQCLGSTFTVTMKEAWTQLYGAVVQAMSRGWNGE------
Platypus	FLDHIRKVMLVIDAAVIHLDDLSSLEEYLTNLGRKHKAIGVKLSSFST----VGESLLFMLEKCLGPAFSPATREAWTRLYTAMVHAMSRGWGGE------
Zebrafish	FLEHVTKVMLVIDAAVSHLDDLHTLEDFLLNLGRKHQAVGVNTQSFAL----VGESLLYMLQSSLGPAYTTSLRQAWLTMYSIVVSAMTRGWAKNGEHKSN
Fugu	FLEHVTKVMLVIDAAVSHLDDLHSLEDFLLNLGRKHQAVGVNPQSFAT----VGESLLYMLQCSLGQAYTASLRQAWLNMYSVVVAAMSRGWAKNGEDKAD

Supplementary Figure S3. Sequence alignments among mammalian and fish Ngb proteins. Multiple sequence alignment was performed by Clustal W with manual adjustments. Gaps in the sequences are indicated by dashes. Numbers above the sequences correspond to those of the residues of human Ngb.
